# Supplementary material for: Using Videos to Teach Medical Learners How to Address Common Breastfeeding Problems
Source: MedEdPORTAL. 2021 Apr 1;17:11136. doi: 10.15766/mep_2374-8265.11136 (PMC8015641; doi:10.15766/mep_2374-8265.11136)
Supplement: Supplementary file 1 — Instructor Guide.docxBABA Test.docxKnowledge Test.docxSore Nipples Checklist.docxJaundice Checklist.docxPerceived Low Milk Supply Checklist.docxSore Nipples.mp4Jaundice.mp4Perceived Low Milk Supply.mp4Knowledge Test Answers.docxSore Nipples Checklist Answers.pdfJaundice Checklist Answers.pdfPerceived Low Milk Supply Checklist Answers.pdf [file mep_2374-8265.11136-s001.zip › A. Instructor Guide.docx]

Appendix A: Instructor’s Guide

This intervention was designed to improve learners’ knowledge, comfort and confidence in identifying and addressing common breastfeeding problems through three videos on the following topics: Sore Nipples, Jaundice, and Perceived Low Milk Supply. Instructors who are interested in implementing this educational activity have two options available. They can either show the three videos synchronously as part of a formal teaching setting or allow learners to asynchronously watch the videos on their own time for more flexibilty. If the videos are shown together in the formal setting, watching and discussing them will take approximately 90 minutes. However, if learners watch the videos on their own, the educational activity would take approximately 70 minutes.

Step 1: Instructors should have the learners complete two pre-assessments (Appendix B and C) to assess their current comfort, confidence and knowledge of breastfeeding, which should take approximately 15 minutes to complete:

1. Appendix B: 5 questions using the Breastfeeding Attitudes and Behavior Assessment (BABA) to measure the learner's self-perceived confidence and comfort assisting breastfeeding mothers prior to the video education.

2. Appendix C: 20-questions assessing learners’ breastfeeding knowledge prior to video curriculum experience.

Step 2: Once the pre-assessments are complete, instructors should make sure that learners have the corresponding checklists as a means to guide learners’ observations while watching the breastfeeding videos (Appendix G, H and I). The total amount of time needed to watch the videos using the checklists (Appendix D, E, and F) is 40 minutes. However, if instructors want to have a discussion with learners in between watching each of the videos, this may add an additional 10 minutes or more in between each video for a total of 30 minutes.

1. Provide each learner with checklist for the video on sore nipples (Appendix D) to guide their observations while watching the corresponding video. Show them the video (Appendix G- Sore Nipples caused by inadequate latch - 15:11 min) and instruct learners to identify whether they see the behaviors outlined on the checklist being enacted in the video. If the mother offers the information but the physician doesn’t specifically ask, that behavior should not be checked off. The instructors will be provided with an answer key checklist (Appendix K). If instructors have time after the video is shown, engage the learners in a discussion on what they saw in the video, what they learned, and what they would like to know more about. Since the key teaching of Sore Nipples is that generally this is caused by a poor latch, that is the main focus. Concentrate the discussion on the following:

- - What is most common cause of sore nipples
  - Characteristics of professionalism
  - Key historical questions to glean cause of the sore nipples
  - Physical features of a good versus poor latch
  - Some ways to fix a poor latch
  - Anticipatory guidance and future management

Use the checklist as a resource for the answers to these questions.

2. Provide each learner with the checklist for the video on jaundice (Appendix E) to guide their observations while watching the linking video . Show them the video (Appendix H- Jaundice caused by poor milk transfer - 11:31 min), and instruct them to identify whether they see behaviors outlined on the checklist being enacted in the video. The instructors will be provided with an answer key checklist (Appendix L). Again, if instructors have time, they should provide learners with the option of having a discussion based on their observations. The key teaching point is that though initially breastfeeding was not going well, it seems to be going better at the hospital follow up visit with good ouput and improvement of latch. Focus discussion on:

- - Taking a history with open ended questions
  - Getting a detailed history about breastfeeding to assess appropriate intake volume and growth
  - Discuss visual cues to look for to ensure latch is adequate and other ways to assess for good milk transfer including auditory clues
  - Anticipatory guidance and management plans

Use the checklist as a resource for the answers to these questions.

3. Provide each learner with the checklist for perceived low milk supply (Appendix F) to guide their observations while watching the matching video. Show them the video (Appendix I- Perceived low milk supply caused by low maternal confidence - 9:43 min), and instruct them to identify whether they see behaviors outlined on the checklist being enacted in the video. The instructors will be provided with an answer key checklist (Appendix M). Once more, if instructors have time, they should provide learners with the option of having a discussion based on their observations. The key teaching point is that Mom’s milk supply is adequate but she is lacking confidence. Focus discussion on emphasizing what data is needed to comfortably assess if the baby is indeed growing appropriately while also discussing points to go over with mom to convince her of adequate feeding while also supporting her to increase her confidence. Discuss

- - Taking an open ended history focusing on causes that might potentially decrease mom’s milk supply
  - Discuss formula use and when is appropriate to supplement with formula
  - Discuss normal growth for an infant
  - Discuss normal changes infants and mothers go through in the first several months (I.e. stool less frequently, less breast engorgement, more efficient feeding)
  - Review ways to have the following difficult dicussions
    - encourage mom to continue to breastfeed exclusively when she has low confidence versus
    - support her decision to supplement versus
    - When supplementation is necessary and mom is resistant

Step 3: After learners have used the checklists (Appendix D, E, F) to assess the breastfeeding videos (Appendix G, H, I), they should be given the two post-assessments (Appendix B and C) to assess changes in comfort, confidence, and knowledge. These assessments should take approximately 15 minutes. Appendix J is the knowledge test with the correct answers written in bold.
