# Supplementary material for: Using Videos to Teach Medical Learners How to Address Common Breastfeeding Problems
Source: MedEdPORTAL. 2021 Apr 1;17:11136. doi: 10.15766/mep_2374-8265.11136 (PMC8015641; doi:10.15766/mep_2374-8265.11136)
Supplement: Supplementary file 1 — Instructor Guide.docxBABA Test.docxKnowledge Test.docxSore Nipples Checklist.docxJaundice Checklist.docxPerceived Low Milk Supply Checklist.docxSore Nipples.mp4Jaundice.mp4Perceived Low Milk Supply.mp4Knowledge Test Answers.docxSore Nipples Checklist Answers.pdfJaundice Checklist Answers.pdfPerceived Low Milk Supply Checklist Answers.pdf [file mep_2374-8265.11136-s001.zip › B. BABA Test.docx]

**Breastfeeding Attitudes and Behavior Assessment (BABA)**

Please answer according to your experience.

| **How confident are you of your ability to…** |  |  |  |  |  |
| --- | --- | --- | --- | --- | --- |
|  | **Not at all                            Very** | | | | |
| Adequately address parents’ concerns about breastfeeding? | 1 | 2 | 3 | 4 | 5 |
| Find out if a medicine is safe to use while breastfeeding? | 1 | 2 | 3 | 4 | 5 |
| Help a new mother breastfeed her infant? | 1 | 2 | 3 | 4 | 5 |
| **How do you feel about the following?** |  |  |  |  |  |
| I would be comfortable if a mother breastfed in front of me | 1 | 2 | 3 | 4 | 5 |
| I would be comfortable helping a mother breastfeed her baby | 1 | 2 | 3 | 4 | 5 |
