# Supplementary material for: Using Videos to Teach Medical Learners How to Address Common Breastfeeding Problems
Source: MedEdPORTAL. 2021 Apr 1;17:11136. doi: 10.15766/mep_2374-8265.11136 (PMC8015641; doi:10.15766/mep_2374-8265.11136)
Supplement: Supplementary file 1 — Instructor Guide.docxBABA Test.docxKnowledge Test.docxSore Nipples Checklist.docxJaundice Checklist.docxPerceived Low Milk Supply Checklist.docxSore Nipples.mp4Jaundice.mp4Perceived Low Milk Supply.mp4Knowledge Test Answers.docxSore Nipples Checklist Answers.pdfJaundice Checklist Answers.pdfPerceived Low Milk Supply Checklist Answers.pdf [file mep_2374-8265.11136-s001.zip › D. Sore Nipples Checklist.DOCX]

Case 1- Sore Nipples

**Instructions:** Please indicate whether the resident completed the following 10 behaviors by selecting **YES** or **No.** If the standardized patient mother initiates one of these behaviors and the resident acknowledges the patient and follows-up accordingly, then indicate **YES** for that behavior**.** If the resident does not acknowledge or does not address a behavior even when the mom initiates, mark **No.**

**Learner name:________________________________** **Date:_____________________**

| Yes | No |  |
| --- | --- | --- |
| **□** | **□** | ***Opening the interview****:*  **Greeting**  □Acknowledges mom by looking in eyes  □ Doctor introduces self to mother  □ Addresses with conversation skill  □ Looks relaxed (Sits or stands in relaxed pose) |
|  |  | ***History****:* |
| **□** | **□** | **Gathers history with open ended questions**  □ Listens to mother’s answers  □ Asks mother to talk about her reasons for breastfeeding  □ Asks what mother’s goals are for breastfeeding  □ Assesses social support at home  □ Assesses breastfeeding support  □ Asks if baby has been rooming in or spending time in nursery  □ Physician discusses that baby is preterm (37 weeks) and this may affect latch  □ Physician discusses that NG suction may affect feeds negatively  □ Assesses output  □ Urine □ Stool |
| **□** | **□** | **Asks about feeds:**  □ Frequency  □ Exclusivity  □ If not exclusive, what was used to give formula/solids? Spoon, cup, syringe, SNS, bottle  □ Pacifiers |
| **□** | **□** | **Physician questions about pain during feed**  □ Assesses when the pain occurs during the feed (beginning vs whole feed)  □ Where is the pain  □ Assesses severity of pain  □ What makes pain better/worse |

| Yes | No |  |
| --- | --- | --- |
| **□**  **□** | **□**  **□** | ***Physical Exam:***  **Asks to examine breast/nipple to assess for damage**  □ Washes hands  □ Looks in **baby’s** mouth for thrush/teeth/tongue tie  **Watches breastfeeding ** if baby asleep- resident should talk through what he is looking/for and assessing**  □ Assesses baby’s positioning  □ Tummy to tummy  □ Assesses mother’s positioning and comfort  □ Recommends not leaning over  □ Recommends to bring baby to breast  □ Assesses mother for tight shoulders  □ Assesses for anxiety |
| **□** | **□** | **Assesses latch**  □ Shows mom how to express colostrum  □ Shows mother how to touch nipple to nose to get baby to open mouth  □ Discusses importance of wide open mouth and not pinched  □ Shows sandwiching the areola to get better latch  □ Teaches Deep compression (C or U hold) to help increase milk  ejection effect (fingers parallel to lips)  □ Assesses for clicking or noises that indicate a poor latch  □ Assesses mom’s comfort or pain level  □ Explains asymmetric latch (more underside areola/ more than nipple)  □ Ensures nose not buried, elbow push of baby’s bottom  □ Listens for swallowing (counts suck:swallow ratio)  □ Teaches mom to listen for swallowing  □ Watches for a pause (swallow) or drop in jaw  □ Assesses how does mom removes the baby from breast -break suction |
| **□** | **□** | **Resident gives feedback to mom about what he visually sees during the feed that is good and what needs adjusting** |
|  |  |  |
|  |  | ***Plan****:* |
| **□** | **□** | **Provides encouragement for mom**  □ Guides mother and empowers her to make a plan that she is comfortable with |
| **□** | **□** | **Gives instructions for future management depending on assessment**  □ Links patient to community breastfeeding support  □ Gives resource handout  □ Physician has mom repeat back what the plan is |
