# Supplementary material for: Using Videos to Teach Medical Learners How to Address Common Breastfeeding Problems
Source: MedEdPORTAL. 2021 Apr 1;17:11136. doi: 10.15766/mep_2374-8265.11136 (PMC8015641; doi:10.15766/mep_2374-8265.11136)
Supplement: Supplementary file 1 — Instructor Guide.docxBABA Test.docxKnowledge Test.docxSore Nipples Checklist.docxJaundice Checklist.docxPerceived Low Milk Supply Checklist.docxSore Nipples.mp4Jaundice.mp4Perceived Low Milk Supply.mp4Knowledge Test Answers.docxSore Nipples Checklist Answers.pdfJaundice Checklist Answers.pdfPerceived Low Milk Supply Checklist Answers.pdf [file mep_2374-8265.11136-s001.zip › E. Jaundice Checklist.DOCX]

Case 2- Jaundice

**Instructions:** Please indicate whether the resident completed the following 10 behaviors by selecting **YES** or **No.** If the standardized patient mother initiates one of these behaviors and the resident acknowledges the patient and follows-up accordingly, then indicate **YES** for that behavior**.** If the resident does not acknowledge or does not address a behavior even when the mom initiates, mark **No.**

**Learner name:________________________** **Date:_____________________**

| Yes | No |  |
| --- | --- | --- |
| **□** | **□** | ***Opening the interview****:*  **Greeting**  □Acknowledges mom by looking in eyes  □ Doctor introduces self to mother  □ Addresses with conversation skill  □ Looks relaxed (Sits or stands in relaxed pose) |
| **□**  **□** | **□**  **□** | ***Data Collection****:*  **Gathers history with open ended questions**  □ Listens to mother’s answers  □ Asks mother to talk about her reasons for breastfeeding  □ Asks what mother’s goals are for breastfeeding  □ Assesses social support at home  □ Assesses breastfeeding support    **Asks about feeds:**  □ Frequency  □ Length of feed  □ Assesses how feeding begins  □ Baby led (mother notices feeding cues)  □ Mother led (scheduled)  □ How does feeding end  □ Baby led (comes off breast on own or falls asleep)  □ Mother led (breaks suction)  □ Physician questions about pain during feed  □ Assesses when the pain occurs during the feed (beginning or the whole time  □ Exclusivity  □ If not exclusive, what was used to give formula? Spoon, cup, syringe, SNS, bottle  □ Asks about supplementation (any at this point is a red flag)  □ If supplementing, how much and why  □ Pacifiers  □ Term/preterm (baby is term)  □ Asks does mom hear swallowing |
| Yes | No |  |
| **□**  **□** | **□**  **□** | ***Data Collection, continued****:*  **Assesses output**  □ Urine □ Stool (number and color)  **Assesses weight and calculates % loss** |
| **□**  **□**  **□** | **□**  **□**  **□** | ***Physical Exam****:*  **Watches breastfeeding ** if baby asleep- resident should talk through what he is looking/for and assessing**  □ Asks to examine breast and/or breastfeeding  □ Looks in **baby’s** mouth for thrush/teeth/tongue tie  □ Washes hands  □ Assesses baby’s positioning  □ Tummy to tummy  □ Assesses mother’s positioning and comfort  □ Recommends not leaning over  □ Recommends to bring baby to breast  □ Assesses mother for tight shoulders  □ Assesses for anxiety  **Assesses latch**  □ Shows mom how to express colostrum  □ Shows mother how to touch nipple to nose to get baby to open mouth  □ Discusses importance of wide open mouth and not pinched  □ Shows sandwiching the areola to get better latch  □ Teaches Deep compression (C or U hold) to help increase milk ejection effect (fingers parallel to lips)  □ Assesses for clicking or noises that indicate a poor latch  □ Assesses mom’s comfort or pain level  □ Explains asymmetric latch (more underside areola/ more than nipple)  □ Ensures nose not buried, elbow push of baby’s bottom  **Assesses swallowing**  □ Listens for swallowing (counts suck:swallow ratio)  □ Teaches mom to listen for swallowing ensuring milk transfer  □ Watches for a pause (swallow) or drop in jaw  □ Assesses how does mom removes the baby from breast -break suction |
| **□** | **□** | ***Plan****:*  **Provides encouragement for mom**  □ Guides mother and empowers her |
|  |  |  |
| Yes | No |  |
| **□** | **□** | ***Plan, Continued:***  **Gives instructions for future management and resources**  □ Recommends to increase frequency feeds (q2 day, q3 night)  □ Discusses pumping to get breastmilk to increase amount offered if needed  □ If Recommends to give extra milk then by cup/syringe/spoon (may not need supplement)  □ Assesses need for bilirubin level  □ Links patient to community breastfeeding support  □ Makes follow up plans in near future to check weight, etc  □ Gives specific recommendations on expected output  □ If trouble waking baby, recommends skin to skin  □ Physician has mom repeat back what the plan is |
