# Supplementary material for: Using Videos to Teach Medical Learners How to Address Common Breastfeeding Problems
Source: MedEdPORTAL. 2021 Apr 1;17:11136. doi: 10.15766/mep_2374-8265.11136 (PMC8015641; doi:10.15766/mep_2374-8265.11136)
Supplement: Supplementary file 1 — Instructor Guide.docxBABA Test.docxKnowledge Test.docxSore Nipples Checklist.docxJaundice Checklist.docxPerceived Low Milk Supply Checklist.docxSore Nipples.mp4Jaundice.mp4Perceived Low Milk Supply.mp4Knowledge Test Answers.docxSore Nipples Checklist Answers.pdfJaundice Checklist Answers.pdfPerceived Low Milk Supply Checklist Answers.pdf [file mep_2374-8265.11136-s001.zip › F. Perceived Low Milk Supply Checklist.DOCX]

Case 3- Perceived Low Milk Supply

**Instructions:** Please indicate whether the resident completed the following 10 behaviors by selecting **YES** or **No.** If the standardized patient mother initiates one of these behaviors and the resident acknowledges the patient and follows-up accordingly, then indicate **YES** for that behavior**.** If the resident does not acknowledge or does not address a behavior even when the mom initiates, mark **No.**

**Learner name:____________________________** **Date:_____________________**

| Yes | No |  |
| --- | --- | --- |
| **□** | **□** | ***Opening the interview****:*  **Greeting**  □ Acknowledges mom by looking in eyes  □ Doctor introduces self to mother  □ Addresses with conversation skill  □ Looks relaxed (Sits or stands in relaxed pose) |
| **□**  **□** | **□**  **□** | ***Data Collection****:*  **Gathers history with open ended questions**  □ Listens to mother’s answers  □ Asks mother to talk about her reasons for breastfeeding  □ Assesses mother’s goals for breastfeeding  □ Asks does she have prior experience with breastfeeding  □ Assesses social support at home  □ Assesses breastfeeding support  **Asks why she thinks milk supply is low**  □ Asks when she thinks the milk supply became a problem  □ Assesses whether or not she has gone back to work/school  □ Birth control when and what mode  □ Did she have breast changes during pregnancy (tubular breasts)  □ Asks about any past breast surgeries  □ Asks about other meds mom is taking  □ Smoking  □ Alcohol |
|  |  |  |
|  |  |  |
| Yes | No |  |
| **□** | **□** | ***Data collection, continued:***  **Asks about breastfeeds:**  □ Frequency  □ Assesses how feeding begins  □ Baby led (mother notices feeding cues)  □ Mother led (scheduled)  □ Assesses how does feeding end  □ Baby led (comes off breast on own or falls asleep)  □ Mother led (breaks suction)  □ Assesses if mother hears swallowing  □ Assesses output  □ Urine  □ Stool |
| **□**  **□** | **□**  **□** | **Asks about formula use**  □ Asks why she wants to start formula  □ If already supplemented with formula, asks  □ When did supplementation start  □ Why  □ How much  □ How often  □ Discusses why not to use formula (if decrease demand then decrease supply- physiology)  **Reassures mother with growth chart (fact)**  □ That baby is gaining weight  □ Explains growth chart  □ Educates that body adjusts and breasts do not get engorged as they did earlier |
| **□**  **□** | **□**  **□** | ***Physical Exam****:*  **Watches breastfeed ** if baby asleep- resident should talk through what he is looking/for and assessing**  □ Looks in **baby’s** mouth for thrush/teeth/tongue tie  □ Washes hands  □ Asks permission to watch breastfeed  □ Listens for swallowing or look for the pause or drop in jaw  □ Teaches deep compressions  □ Gives suggestions if appropriate  **Reassures, encourages, and gives feedback about adequate latch and milk transfer**  □ Asks again if any concerns |
|  |  |  |
| Yes | No |  |
| **□**  **□** | **□**  **□** | ***Plan****:*  **Provides encouragement for mom**  □ Guides mother but empowers her to make a breastfeeding plan that she is comfortable with  **Gives instructions for future management**  □ Mainly dealing with formula supplementation in the future  □ Links patient to community breastfeeding support and resources  □ Gives handout  □ Physician makes a weight check appointment in near future  □ Physician has mom repeat back what the plan is |
