# Supplementary material for: Using Videos to Teach Medical Learners How to Address Common Breastfeeding Problems
Source: MedEdPORTAL. 2021 Apr 1;17:11136. doi: 10.15766/mep_2374-8265.11136 (PMC8015641; doi:10.15766/mep_2374-8265.11136)
Supplement: Supplementary file 1 — Instructor Guide.docxBABA Test.docxKnowledge Test.docxSore Nipples Checklist.docxJaundice Checklist.docxPerceived Low Milk Supply Checklist.docxSore Nipples.mp4Jaundice.mp4Perceived Low Milk Supply.mp4Knowledge Test Answers.docxSore Nipples Checklist Answers.pdfJaundice Checklist Answers.pdfPerceived Low Milk Supply Checklist Answers.pdf [file mep_2374-8265.11136-s001.zip › J. Knowledge Test Answers.docx]

Video knowledge test - **BOLDED CORRECT ANSWERS**

1) Mothers can support an infant’s acquisition of milk from the breast by:

a. Frequent burping

b. Taking breaks during breastfeeding

**c. Deep compression of the breast**

d. Holding infant’s head deeply compressed to the breast

e. Maintaining a high calorie diet while breastfeeding

2) In addition to stabilizing an infant’s heart rate, respiratory rate, and temperature,

skin to skin contact after delivery also stabilizes an infant’s:

a. Serum bicarbonate

**b. Serum glucose**

c. Serum bilirubin

d. Serum sodium

e. Serum potassium

3) The most effective and appropriate way to break an infant’s latch is to:

a. Tilt the infant’s head back away from the nipple

b. Stroke the infant’s cheek

**c. Insert a finger into the infant’s mouth to break the suction**

d. Wait for a pause in the infant’s suck/swallow routine and pull away

e. Pull baby off with gentle pressure

4) Sore nipples or prolonged pain during breastfeeding are concerning because they indicate:

a. Inverted nipples

b. Inadequate milk supply

c. Loss of milk supply

d. Poor suck and swallow technique

**e. Poor latch**

5) In order to identify potential problems with breastfeeding, assessment of an

infant’s mouth should include all of the following EXCEPT:

a. Short tongue frenulum

b. Intact palate

c. Thrush

**d. Gum alignment**

e. Presence of teeth

6) Late preterm infants are predisposed to problems with breastfeeding and

subsequently jaundice due to:

1. **Difficulty establishing latch with good milk transfer and less mature livers than term infants**

b. Difficulty stabilizing temperature and glucose

c. Difficulty establishing breastfeeding within 30 minutes of delivery since they usually go to the NICU

d. Difficulty passing meconium and more frequent spit ups

e. Difficulty maintaining normal newborn sleep and wake cycles

7) All of the following are early indicators of hunger in a newborn EXCEPT:

**a. Crying**

b. Rooting

c. Sucking on fingers

d. A quiet wakeful state

e. Lip smacking with tongue protrusion

8) Establishment of maternal milk supply is promoted through:

1. Skin to skin contact
2. Adequate sleep
3. Frequent breastfeeding
4. High calorie diet
5. **A and C**

9) Breastfeeding neonates should be fed:

- 1. **8 to 12 times during a 24hr period**
  2. Only when awake
  3. When baby gives hunger cues
  4. During sensations of breast fullness
  5. After every wet diaper

10) Signs of adequate milk intake are

1. no weight loss after day 5
2. at least 4 yellow stools a day after day 4
3. a baby that falls asleep at the breast after rhythmic swallowing heard for 5-10 minutes
4. B and C
5. **A, B, and C**

11) The recommended birth control for breastfeeding women is

a. estrogen only OCP

**b. progesterone only OCP or IM**

c. estrogen and progesterone combination

d. breastfeeding is protective enough and no additional birth control is

needed

e. oral contraceptives are contraindicated during breastfeeding

12) A mom who is concerned about her milk supply can be reassured by

1. seeing and having her child’s growth chart explained to her
2. having a breastfeeding session observed by a professional
3. discussing her concerns and addressing each concern
4. A and B
5. **All the above**

13) Ways to help encourage a good latch are:

1. not feeding the baby until milk let down occurs
2. have mom crouch over baby ensuring skin to skin
3. use the sandwich hold to ensure a deeper latch
4. wait for the baby to have a wide open mouth
5. **C and D**

14) Hypoglycemia, both symptomatic and asymptomatic, is a common concern in healthy term breastfed neonates. While glucose monitoring should be performed only in high-risk infants and those who are symptomatic, the management strategies employed to prevent and treat hypoglycemia should support breastfeeding. Which of

the of the following strategies is the BEST method to prevent symptomatic hypoglycemia:

a. Glucose monitoring every thirty minutes following delivery

b. Oral glucose solution by mouth immediately following birth, followed by

breastfeeding on demand.

**c. Early initiation of breastfeeding on demand, within 30-60 minutes after**  **delivery**

d. Supplement with formula if baby found to be hypoglycemic (glucose less than 45 mg/dL (<2.5 mmol/L)

e. All of the above

15) All of the following are recommended to encourage successful breastfeeding

EXCEPT:

a. Initiation of breastfeeding within one hour of birth

b. Avoiding the use of pacifiers and artificial nipples in term breastfeeding infants

c. Continuous rooming in with breastfeeding on demand

**d. Restricting length of breastfeeding time to prevent nipple soreness and**  **engorgement**

e. Avoiding use of supplemental formula during the early stages of milk production

16) A mother with a three-day old baby presents with sore nipples. The problem began with the first feeding and has persisted with every feeding. The most likely source of the problem is:

1. Baby’s suck is too strong
2. Feeding time is too long
3. Lack of nipple preparation during pregnancy
4. Inverted nipples
5. **Poor attachment to the breast**

17) Hospital policies that interfere with breastfeeding include all of the following **EXCEPT**:

1. Moving the infant to the nursery for the night to allow mother to rest and build up her milk supply
2. Feedings scheduled every 4 hours to allow mother’s breasts to make more milk
3. Use of pacifiers to prevent the infant using mother as a pacifier and giving her sore nipples
4. **Showing all mothers how to express or pump breastmilk in case they are separated from their infants**
5. Routine water supplementation by dropper to prevent dehydration
6. In which of the following circumstances should it be necessary to delay the initiation of breastfeeding after delivery (for more than 1 hour):
7. C-section with spinal anesthesia
8. Mother fatigued due to a long and difficult labor
9. Mother receiving MgSO4 for preeclampsia
10. All of the above
11. **None of the above**
12. An acceptable medical reason to supplement a breastfed infant in the hospital is:
13. To quiet a fussy baby
14. **Separation from the mother due to maternal or infant illness**
15. To teach the baby to take a bottle for later
16. To prevent dehydration
17. To allow the mother to rest
18. When women believe they have a low milk supply, they can **BEST** be helped by:
19. Supplementing the baby by cup or finger-feeding to give the mother a break
20. **Taking a breastfeeding history and assessing the infant at the breast**
21. Having the mother pump for 15 minutes after every feeding
22. Teaching the mother to assess urine output
23. Reassurance that it is only an infant growth spurt – that her milk supply is adequate
