# Supplementary material for: Using Videos to Teach Medical Learners How to Address Common Breastfeeding Problems
Source: MedEdPORTAL. 2021 Apr 1;17:11136. doi: 10.15766/mep_2374-8265.11136 (PMC8015641; doi:10.15766/mep_2374-8265.11136)
Supplement: Supplementary file 1 — Instructor Guide.docxBABA Test.docxKnowledge Test.docxSore Nipples Checklist.docxJaundice Checklist.docxPerceived Low Milk Supply Checklist.docxSore Nipples.mp4Jaundice.mp4Perceived Low Milk Supply.mp4Knowledge Test Answers.docxSore Nipples Checklist Answers.pdfJaundice Checklist Answers.pdfPerceived Low Milk Supply Checklist Answers.pdf [file mep_2374-8265.11136-s001.zip › K. Sore Nipples Checklist Answers.pdf]

Case 1- Sore Nipples

Instructions: Please indicate whether the video demonstrated the following 10 behaviors by selecting **YES** or **No**. If the information is provided by mom without being asked, mark **No.**

Learner name: ______________________________ **Date:** ______________

Opening the interview

Greeting

- [x] Acknowledges mom by looking in eyes
- [x] Introduces self to mother
- [x] Addresses with conversation skill
- [x] Looks relaxed (sits or stands in relaxed pose)

History

Gathers history with open ended questions

- [x] Listens to mother's answers
- [x] Asks mother to talk about her reasons for breastfeeding
- [ ] Asks what mother's goals are for breastfeeding
- [x] Assesses social support at home
- [x] Assesses breastfeeding support
- [ ] Asks if baby has been rooming in or spending time in nursery
- [x] Educates that baby is preterm and this may affect latch
- [x] Discusses the NG suction may affect feeds negatively
- [x] Assesses output (urine/stool)

Asks about feeds

- [x] Frequency
- [x] Exclusivity
    - [ ] If not exclusive, what was used to give formula/solids? Spoon, cup, syringe, SNS, bottle
- [x] Pacifiers

Questions about pain during feed

- [x] Assesses when the pain occurs during feed (beginning or whole feed)
- [ ] Asks where is the pain
- [ ] Assesses severity of pain
- [ ] Asks what makes the pain better/worse

Physical Exam

Asks to examine breast/nipple to assess for damage

- [x] Washes hands
- [x] Looks in baby's mouth for thrush/teeth/tongue tie

Watches breastfeeding

- [x] Assesses baby's positioning
  - [x] Tummy to tummy
- [ ] Assesses mother's positioning and comfort
  - [ ] Recommends not leaning over
  - [ ] Recommends to bring baby to breast
  - [x] Assesses mother for tight shoulders
  - [ ] Assesses for anxiety

Assesses latch

- [ ] Shows mom how to express colostrum
- [ ] Shows mother how to touch nipple to nose to get baby to open mouth
- [ ] Discusses importance of wide open mouth and not pinched
- [x] Shows sandwiching the areola to get better latch
- [x] Teaches Deep compression (C or U hold) to help increase milk ejection effect (fingers parallel to lips)
- [ ] Assesses for clicking or noises that indicate a poor latch
- [x] Assesses mom's comfort or pain level
- [ ] Explains asymmetric latch (more underside areola/ more than nipple)
- [x] Ensures nose not buried, elbow push of baby's bottom
- [x] Listens for swallowing (counts suck: swallow ratio)
- [x] Teaches mom to listen for swallowing
- [x] Watches for a pause (swallow) or drop in jaw
- [x] Assesses how does mom removes the baby from breast -break suction
- [x] Gives feedback on what is seen visually –what is good and adjustments needed

Plan:

Provides encouragement for mom

- ☐ Guides mother and empowers her to make a plan with which she is comfortable

Gives instructions for future management depending on assessment

- ☐ Links patient to community breastfeeding support
- ☑ Gives resource handout
- ☐ Has mom repeat back what the plan is
