# Supplementary material for: Using Videos to Teach Medical Learners How to Address Common Breastfeeding Problems
Source: MedEdPORTAL. 2021 Apr 1;17:11136. doi: 10.15766/mep_2374-8265.11136 (PMC8015641; doi:10.15766/mep_2374-8265.11136)
Supplement: Supplementary file 1 — Instructor Guide.docxBABA Test.docxKnowledge Test.docxSore Nipples Checklist.docxJaundice Checklist.docxPerceived Low Milk Supply Checklist.docxSore Nipples.mp4Jaundice.mp4Perceived Low Milk Supply.mp4Knowledge Test Answers.docxSore Nipples Checklist Answers.pdfJaundice Checklist Answers.pdfPerceived Low Milk Supply Checklist Answers.pdf [file mep_2374-8265.11136-s001.zip › L. Jaundice Checklist Answers.pdf]

Case 2- Jaundice

Instructions: Please indicate whether the video demonstrated the following 10 behaviors by selecting **YES** or **No**. If the information is provided by mom without being asked, mark **No**.

Learner name: ______________________________ **Date:** ______________

Opening the interview

Greeting

- ☑ Acknowledges mom by looking in eyes
- ☑ Introduces self to mother
- ☑ Addresses with conversation skill
- ☑ Looks relaxed (sits or stands in relaxed pose)

History

Gathers history with open ended questions

- ☑ Listens to mother's answers
- ☐ Asks mother to talk about her reasons for breastfeeding
- ☐ Asks what mother's goals are for breastfeeding
- ☐ Assesses social support at home
- ☐ Assesses breastfeeding support

Asks about feeds

- ☑ Frequency
- ☑ Length of feed
- ☐ Assesses how feeding begins
  - ☐ Baby led (mother notices feeding cues)
  - ☐ Mother led (scheduled)
- ☐ How does feeding end
  - ☐ Baby led (comes off breast on own or falls asleep)
  - ☐ Mother led (breaks suction)
- ☑ Physician questions about pain during feed
- ☑ Assesses when the pain occurs during the feed (beginning or whole feed)
- ☐ Exclusivity
  - ☐ If not exclusive, what was used to give formula/solids? Spoon, cup, syringe, SNS, bottle
  - ☐ Asks about supplementation (any at this point is a red flag)

- ☐ If supplementing, how much and why
- ☐ Pacifiers
- ☐ Term/preterm
- ☑ Asks if mom hears swallowing

Assesses output

- ☐ Urine
- ☑ Stool

Growth

- ☑ Assesses weight
- ☐ Calculates % loss

Physical Exam

Watches breastfeeding

- ☐ Washes hands
- ☑ Looks in baby's mouth for thrush/teeth/tongue tie
- ☐ Assesses baby's positioning
  - ☐ Tummy to tummy
- ☐ Assesses mother's positioning and comfort
  - ☐ Recommends not leaning over
  - ☐ Recommends to bring baby to breast
  - ☐ Assesses mother for tight shoulders
  - ☐ Assesses for anxiety

Assesses latch

- ☐ Shows mom how to express colostrum
- ☑ Shows mother how to touch nipple to nose to get baby to open mouth
- ☑ Discusses importance of wide open mouth and not pinched
- ☐ Shows sandwiching the areola to get better latch
- ☐ Teaches Deep compression (C or U hold) to help increase milk ejection effect (fingers parallel to lips)
- ☐ Assesses for clicking or noises that indicate a poor latch
- ☑ Assesses mom's comfort or pain level
- ☐ Explains asymmetric latch (more underside areola/ more than nipple)
- ☐ Ensures nose not buried, elbow push of baby's bottom

Assesses swallowing

- ☐ Listens for swallowing (counts suck: swallow ratio)

- ☐ Teaches mom to listen for swallowing ensuring milk transfer
- ☐ Watches for a pause (swallow) or drop in jaw
- ☐ Assesses how does mom removes the baby from breast -break suction

Plan:

Provides encouragement for mom

- ☑ Guides mother and empowers her to make a plan with which she is comfortable

Gives instructions for future management depending on assessment

- ☐ Recommends to increase frequency of feeds to q2 day, q3 night
- ☐ Discusses pumping to help increase breastmilk volume if needed
- ☑ Assesses need for bilirubin level
- ☑ Gives specific recommendations on expected output
- ☐ Counsels if trouble waking baby, recommend skin to skin
- ☑ Makes follow up plans in near future to check weight, etc
- ☑ Links patient to community breastfeeding support
- ☐ Gives resource handout
- ☐ Has mom repeat back what the plan is
