# Supplementary material for: Using Videos to Teach Medical Learners How to Address Common Breastfeeding Problems
Source: MedEdPORTAL. 2021 Apr 1;17:11136. doi: 10.15766/mep_2374-8265.11136 (PMC8015641; doi:10.15766/mep_2374-8265.11136)
Supplement: Supplementary file 1 — Instructor Guide.docxBABA Test.docxKnowledge Test.docxSore Nipples Checklist.docxJaundice Checklist.docxPerceived Low Milk Supply Checklist.docxSore Nipples.mp4Jaundice.mp4Perceived Low Milk Supply.mp4Knowledge Test Answers.docxSore Nipples Checklist Answers.pdfJaundice Checklist Answers.pdfPerceived Low Milk Supply Checklist Answers.pdf [file mep_2374-8265.11136-s001.zip › M. Perceived Low Milk Supply Checklist Answers.pdf]

Case 3- Perceived Low Milk Supply

Instructions: Please indicate whether the video demonstrated the following 10 behaviors by selecting **YES** or **No**. If the information is provided by mom without being asked, mark **No**.

Learner name: ______________________________ **Date:** ______________

Opening the interview

Greeting

- [x] Acknowledges mom by looking in eyes
- [x] Introduces self to mother
- [x] Addresses with conversation skill
- [x] Looks relaxed (sits or stands in relaxed pose)

History

Gathers history with open ended questions

- [x] Listens to mother's answers
- [ ] Asks mother to talk about her reasons for breastfeeding
- [x] Asks what mother's goals are for breastfeeding
- [ ] Asks if she has prior experience with breastfeeding
- [ ] Assesses social support at home
- [ ] Assesses breastfeeding support

Asks why she thinks her milk supply is low

- [x] Asks when she thinks the milk supply became a problem
- [ ] Assesses whether or not she has gone back to work/school
- [x] Birth control when and what mode
- [x] Did she have breast changes during pregnancy (tubular breasts)
- [x] Asks about past breast surgeries
- [ ] Asks about other meds mom is taking
- [x] Smoking
- [x] Alcohol

Asks about breastfeeds

- [x] Frequency
- [ ] Assesses how feeding begins
  - [ ] Baby led (mother notices feeding cues)
  - [ ] Mother led (scheduled)
- [ ] Assesses how feeding ends

- ☐ Baby led (comes off breast on own or falls asleep)
  - ☐ Mother led (breaks suction)
- ☑ Assesses if mom hears swallowing
- ☑ Assesses output (urine/stool)

Asks about formula use

- ☑ Asks why she wants to start formula
- ☐ If already supplemented with formula, asks
  - ☐ When did supplementation start
  - ☐ Why
  - ☐ How much
  - ☐ How often
- ☐ Discusses why not to use formula (decreased demand decreases supply)

Reassured mother with growth chart

- ☑ Explains growth chart
- ☑ Shows mom that baby is gaining weight
- ☑ Educates that body adjusts and breasts do not get engorged as they did earlier

Physical Exam

Watches breastfeeding

- ☐ Looks in baby's mouth for thrush/teeth/tongue tie
- ☐ Asks permission to watch a breastfeed
- ☑ Listens for swallowing or looks for the pause or drop in jaw
- ☑ Teaches deep compression
- ☑ Gives suggestions if appropriate
- ☑ Reassures, encourages, and gives feedback

Plan

Provides encouragement for mom

- ☑ Guides mother and empowers her to make a plan with which she is comfortable

Gives instructions for future management

- ☐ Formula supplementation in the future
- ☑ Makes follow up plans in near future to check weight, etc
- ☐ Links patient to community breastfeeding support
- ☐ Gives resource handout
