# Supplementary material for: Using Videos to Teach Medical Learners How to Address Common Breastfeeding Problems
Source: MedEdPORTAL. 2021 Apr 1;17:11136. doi: 10.15766/mep_2374-8265.11136 (PMC8015641; doi:10.15766/mep_2374-8265.11136)
Supplement: Supplementary file 1 — Instructor Guide.docxBABA Test.docxKnowledge Test.docxSore Nipples Checklist.docxJaundice Checklist.docxPerceived Low Milk Supply Checklist.docxSore Nipples.mp4Jaundice.mp4Perceived Low Milk Supply.mp4Knowledge Test Answers.docxSore Nipples Checklist Answers.pdfJaundice Checklist Answers.pdfPerceived Low Milk Supply Checklist Answers.pdf [file mep_2374-8265.11136-s001.zip › _Educational_Summary_Report_11136.pdf]

MedEdPORTAL® The AAMC Journal of Teaching and Learning Resources

Original Publication Open Access

Using Videos to Teach Medical Learners How to Address Common Breastfeeding Problems

Kathryn McLeod, MD, IBCLC*, Jennifer Waller, PhD, Tasha R. Wyatt, PhD

*Corresponding author: ksmcleod@augusta.edu

Abstract

Introduction: Though breast milk is the recommended first food, only 84% of mothers try breastfeeding, and 19% of those babies are supplemented with formula by the second day of life. Mothers often fall short of their breastfeeding goals, which has been traced in part to primary care physicians' discomfort and lack of confidence in assisting breastfeeding mothers. A video-based educational intervention was developed to enhance learner comfort and confidence in supporting breastfeeding. **Methods:** Prior to watching the videos, two separate groups of learners (25 primary care residents and 40 medical students) were given two assessments to assess their baseline knowledge and self-perceived comfort and confidence with breastfeeding based on the American Academy of Pediatrics residency breastfeeding curriculum. We gave the learners a checklist to guide their observations while watching the videos. The learners repeated the assessments after watching the videos. **Results:** Students who participated showed a statistically significant increase in breastfeeding knowledge and self-perceived comfort and confidence in assisting mothers with common breastfeeding problems. Additionally, residents increased their knowledge and confidence, but not their comfort with breastfeeding. **Discussion:** The videos seem to have been most helpful in assisting learners with confidence with helping breastfeeding mothers and knowledge of breastfeeding. The videos also improved student comfort but were less effective in increasing residents' comfort as residents generally self-rated their comfort level as high preceding the video curriculum. These videos provide an effective way to begin sensitizing learners to the importance of breastfeeding, common challenges, and potential solutions for new mothers.

Keywords
Breastfeeding, OB/GYN, Lactation, Clinical Teaching/Bedside Teaching, Self-Regulated Learning, Virtual Learning

Educational Objectives

By the end of this activity, learners will be able to:

1. Develop comfort and confidence in assessing a baby's breastfeeding latch, identifying potential breastfeeding problems, and devising solutions for preventing sore nipples in a breastfeeding mother.
2. Identify visual and audible cues to determine if the baby is taking enough milk, thus preventing neonatal jaundice, and articulate these cues to a breastfeeding mother.
3. Conduct a relevant history to evaluate whether a mother is sufficiently feeding her baby.

Citation:
McLeod K, Waller J, Wyatt TR. Using videos to teach medical learners how to address common breastfeeding problems. *MedEdPORTAL*. 2021;17:11136. https://doi.org/10.15766/mep_2374-8265.11136

Introduction

Breastfeeding has positive benefits for both mothers and babies. While it protects newborns from respiratory and gastrointestinal infections and, later, diabetes and obesity, it also decreases mothers' risk of breast and ovarian cancer, postpartum depression, heart disease, and type 2 diabetes.[1-3] Thus, the general consensus is that physicians should be trained to improve breastfeeding rates in the general population.[4,5] At present, breastfeeding is considered the standard of care for feeding newborns and infants. However, many mothers do not breastfeed or, in some cases, fail to reach their breastfeeding goals. According to the most recent hospital data, only 84% of mothers attempt breastfeeding, and 19% of those babies have been supplemented with formula by the second day of life.[6] Several reasons have been cited, including new mothers' lack of prenatal breastfeeding education, limited family support, and a need to return to school or work, among other personal reasons.[7,8] Research indicates that training primary care residents in breastfeeding education has potential for increasing

Copyright © 2021 McLeod et al. This is an open-access publication distributed under the terms of the Creative Commons Attribution license.

breastfeeding rates. Primary care providers tend to be a mother's first contact after giving birth and offer an important opportunity to ensure new mothers' concerns are met.[9,10]

However, although previous research has shown that, with training, residents feel more confident and comfortable in talking to mothers about breastfeeding, as well as able to improve patient education,[11,12] training residents on how to interact with breastfeeding mothers is challenging. Several reasons have been cited, including that the intimate examinations of mothers' breasts and the observations needed to improve residents' education tend to produce anxiety in learners.[13,14] As a result, medical educators have attempted to create multiple interventions to train residents on breastfeeding. For example, the American Academy of Pediatrics (AAP) has created a set of teaching slides[15] to guide clinicians in helping breastfeeding mothers, medical institutions have created helpful videos to achieve similar goals,[16] and several didactic resources are available in *MedEdPORTAL*.[17,18] To date, the most frequently used interventions for student learners to emulate a patient interview are standardized patient (SP) encounters and objective structured clinical examinations (OSCEs). These methods are effective, but they have limitations and implementation barriers, making them challenging to implement for breastfeeding education.[19-21] For example, OSCEs are complicated by satiated infants who are unable to repeat multiple feeding episodes during the allotted time. Additionally, newborns are the ideal age to work on breastfeeding, but since they grow so quickly, trainers are required to frequently recruit new mother-baby dyads as newborns age. SP encounters have proven to be effective for breast exams,[22] but they require repeated SP encounters each year and/or rotation.[23] Furthermore, these repeated encounters are challenging to coordinate when residents are spread across multiple specialties with different clinical and curriculum schedules. Combined, these challenges have made it arduous for residents to receive adequate breastfeeding education in a clinical environment,[24] even for residents in specialties that frequently interact with both parents and children, such as pediatrics.[25]

To address some of these challenges in training residents to work with breastfeeding mothers, a series of videos in breastfeeding management was developed to create a sustainable, self-paced, and flexible way to educate residents. These videos, created by the first author, are different from videos available elsewhere, such as through the AAP, in that they incorporate checklists so that learners are guided in their observations. Other videos rely on learners to decide what must be paid attention to as they address common challenges among newly breastfeeding mothers. We designed our videos and their accompanying checklists to highlight visual cues and teach proper language to use when addressing maternal concerns.[19,20,26,27]

Methods

Overview and Development

In 2019, we showed the breastfeeding videos to two groups of learners to assess their effectiveness in increasing learners' comfort, confidence, and knowledge regarding breastfeeding. The first group's implementation occurred at an in-person 90-minute conference for pediatrics and family medicine residents. The second group received an asynchronous implementation for third- and fourth-year medical students on rotation within their pediatrics and OB/GYN clerkships. We included students as learners in this modified asynchronous implementation because research suggests that the earlier learners are exposed to breastfeeding education, the more comfortable and confident they will be when interacting with breastfeeding mothers on their rotations.[4,15] In both formats, this was the first time each group was given explicit training in breastfeeding. An overview of the two methods of implementation is detailed in Appendix A.

The preferred method of implementing this intervention was the in-person format because it allowed residents to discuss the video content with each other and gave the instructor time to point out and discuss key points. Unlike the in-person resident group, students were not provided protected time for formal teaching on their rotations and therefore watched the videos on their own time, using the checklists to guide their observations. Students in the asynchronous group observed the videos at their own pace when their clinical duties were light.

In-Person Implementation

In the in-person format, the instructor and pediatric and OB/GYN residents were allotted 90 minutes and a large conference room to learn about breastfeeding. The first 15 minutes of the session was spent administering both the Breastfeeding Attitudes and Behavior Assessment (BABA) test (Appendix B) and the knowledge test (Appendix C) preassessments. The instructor then provided learners with observational checklists (Appendices D-F) to use while watching the corresponding breastfeeding videos (Appendices G-I). The checklists guided learners' observations and directed them to behaviors and characteristics representing a high-level interaction between mother and baby. The checklists were developed by four lactation consultants who were internationally board-certified, which required them to have taken 90 hours of lactation-specific

Copyright © 2021 McLeod et al. This is an open-access publication distributed under the terms of the Creative Commons Attribution license.

education, logged 1,000 hours of lactation clinical practice, and passed a written board exam.

Watching the videos and using the checklists took approximately 45 minutes, as the videos ranged from 10 to 15 minutes each. Each video focused on a specific theme related to typical problems with breastfeeding and featured three actors to emulate a live breastfeeding dyad, including an actual breastfeeding mother and her breastfeeding baby, and a physician. In the sore nipples video, learners recognized mothers' sore nipples and how to improve a baby's latch during feeding (Appendix G). The jaundice video focused on how a baby's poor latch resulted in inadequate feeding and how this might lead to longer-term complications, such as jaundice (Appendix H). The perceived low milk supply video provided an overview of how to handle mothers' perceptions of having a low milk supply and what residents could advise in this situation (Appendix I). To develop the videos, we provided actors with scripts adapted from those previously used in SP cases and resident OSCEs to teach residents how to work with breastfeeding mothers.[18-21]

Once the learners had watched the videos and made their observations on the checklists, the instructor led a short discussion (20 minutes for all videos) on the behaviors and characteristics that should have been noted in the videos. (These behaviors and characteristics are included in the answer keys in Appendices K-M.) This part of the intervention was carried out in a large-group discussion format, and in some cases, peers shared their checklists with each other to note what they might collectively have missed.

After the video viewing and discussion, the instructor administered the BABA test (Appendix B) and knowledge test (Appendix C) again as a postassessment to determine changes in learners' comfort, confidence, and knowledge regarding breastfeeding. This postassessment took an additional 10 minutes.

Asynchronous Implementation

In the asynchronous student group, we gave students the preassessment BABA (Appendix B) and knowledge tests (Appendix C), which they turned in immediately. We then gave them the postassessment knowledge and BABA tests to complete and hand in after they had watched the videos at their own pace while on rotation, either at home or at work, using their own computer. Although the students did not have the benefit of discussion with each other or an instructor, we gave them the checklists as resources to help focus on key behaviors essential for a good breastfeeding history and physical exam. Because this administration of the videos did not incorporate group discussion or reviewing parts of the videos, it required learners' self-directed participation over a shorter period of time, around 70 minutes.

Assessment

The BABA test was adapted from an assessment originally developed by the AAP[5,19] to assess learners' comfort, confidence, and knowledge regarding breastfeeding. The AAP BABA assessment asked respondents to rate their confidence in (1) adequately addressing parent's concerns about breastfeeding, (2) finding out if a medicine is safe to use while breastfeeding, and (3) helping a new mother breastfeed her infant on a 5-point scale (1 = *not at all,* 5 = *very*). We added two additional questions, for a total score of 25, to assess learners' potential embarrassment and comfort with breastfeeding: (1) How comfortable would you be if a mother breastfed in front of you? (2) How comfortable are you in helping a mother breastfeed her baby?

The knowledge test was developed by the instructors and included 20 questions. The answer key is included here as Appendix J.

The pre-/postassessment data from both learner groups were analyzed using SAS 9.4 with a statistical significance of $p < .05$. Descriptive statistics on demographics and correct responses to the BABA from pre- and postassessment were calculated. Changes from pre- to postassessment on correct responses to the BABA items were also assessed using McNemar's test or Bowker's test of symmetry. Additionally, a paired t test was used to examine changes in the BABA score (sum of addressing concerns, medication safety, helping breastfeed, not being embarrassed to breastfeed in front of others, and not being embarrassed to help breastfeed) between the pre- and postassessment.

Results

Students

The demographic breakdown of the group of 40 students consisted of the following: Twenty-five were female (62%), and 15 were male (38%). Racially, three identified as African American (8%), 13 as Asian (32%), 22 as Caucasian (55%), one as Hispanic/Latino (2%), and 1 as other (2%). Tables 1 and 2 give the descriptive statistics and results of differences between students' pre- and posttest knowledge and BABA tests, respectively. Sixty-three percent of students who responded were female; the majority were Caucasian, were in their third year of training, were breastfed as a child, and had no children. For those who did have children, all were breastfed.

Copyright © 2021 McLeod et al. This is an open-access publication distributed under the terms of the Creative Commons Attribution license.

The results indicated that there was an increase from the pre- to posttest in several areas. For example, students increased their knowledge of how to enhance milk intake; effective ways to break a latch; what sore nipples indicate; infant mouth assessment; reasons late preterm infants are predisposed to feeding problems, including jaundice; late hunger signs in infants; when to recommend birth control; and hospital policies that interfere with breastfeeding (Table 1).

The total number of correct responses on the knowledge test significantly increased from pre- to posttest, from 12.9 (95% CI, 12.0-13.7) to 16.7 (95% CI, 16.1-17.3), respectively (Table 1). An analysis of the BABA showed a statistically significant increase in each confidence measure (addressing concerns, medication safety, and helping breastfeed) and in not being embarrassed (comfort) helping mothers breastfeed (Table 2). Additionally, a significant increase in the BABA score from pre- to posttest was seen: from 16.1 (95% CI, 14.9-17.2) to 20.6 (95% CI, 19.6-21.5), respectively, out of a possible 25 points for maximal confidence and comfort with breastfeeding issues (Table 2).

Residents

The demographic breakdown of this group of 25 was as follows: Nineteen were female (76%), and six were male (24%). Racially, five residents identified as Asian (20%), 16 as Caucasian (64%), three as Hispanic/Latino (12%), and one as other (4%). Tables 3 and 4 give the descriptive statistics and results of differences between residents' pre- and posttest knowledge and BABA tests, respectively. Sixty-seven percent of residents who responded were female; the majority were Caucasian, were in their sixth year of training, were breastfed as a child, and had no children. For those who did have children, all were breastfed.

The results indicated that there was an increase from the pre- to posttest in several areas: how to enhance milk intake, effective ways to break a latch, what sore nipples indicate, infant mouth assessment, late hunger signs, baby-friendly concepts, and hospital policies that interfere with breastfeeding (Table 3). The total number of correct responses on the knowledge test also significantly increased from pre- to posttest, from 12.4 (95% CI, 11.5-13.3) to 17.2 (95% CI, 16.5-17.9), respectively (Table 3). For the BABA, statistically significant increases occurred in confidence addressing concerns and confidence in helping breastfeed (Table 4).

However, unlike the changes in scores for students, there was not a significant change in the total BABA score from pre- to posttest for residents (Table 4). In contrast to students, residents rated themselves as being comfortable with breastfeeding mothers before watching the videos. Despite this, there was a significant increase in residents' confidence in addressing mothers and child's breastfeeding concerns.

Table 1. Pre- and Postassessment Knowledge Test Scores for Students ($N = 40$)

	Pre		Post		
**Questionnaire Item**	**No. (%)**	**95% CI for %**	**No. (%)**	**95% CI for %**	***p*[a]**
1. Enhance milk intake	22 (55)	38.9-71.1	38 (95)	87.9-100.0	.0003
2. Skin-to-skin benefits	33 (82)	70.2-94.8	36 (90)	80.3-99.7	.3173
3. Effective way break latch	9 (22)	9.0-36.0	35 (88)	76.8-98.2	<.0001
4. Sore nipples indicate	26 (65)	49.6-80.4	38 (95)	87.9-100.0	.0013
5. Infant mouth assessment	8 (20)	7.0-33.0	32 (80)	67.0-93.0	<.0001
6. Preterm at risk jaundice	25 (62)	46.8-78.1	37 (92)	84.0-100.0	.0027
7. Late hunger sign	5 (12)	1.8-23.2	26 (65)	49.6-80.4	<.0001
8. Milk supply promotion	36 (90)	80.2-99.7	35 (88)	76.8-98.2	.6547
9. Feed schedules	20 (50)	33.8-66.2	25 (62)	46.8-78.2	.0956
10. Adequate milk intake signs	17 (42)	26.5-58.5	18 (45)	28.9-61.1	.7389
11. Recommended birth control	28 (70)	55.1-84.8	37 (92)	84.0-100.0	.0067
12. Confirm adequate milk	40 (100)	NE	36 (90)	80.3-99.7	NE
13. Good latch technique	34 (85)	73.4-96.6	37 (92)	84.0-100.0	.2568
14. Prevent hypoglycemia	28 (70)	55.2-84.8	34 (86)	73.4-96.6	.0578
15. Baby-friendly concepts	34 (85)	73.4-96.6	34 (86)	73.4-96.6	1.0000
16. Sore nipple source	36 (90)	80.3-99.7	38 (95)	87.9-100.0	.4142
17. Hospital policy interfere breastfeeding	27 (68)	52.3-82.6	33 (82)	70.2-94.8	.0339
18. Delay breastfeeding initiation	25 (62)	46.8-78.2	23 (58)	41.5-73.5	.3173
19. When to supplement	31 (78)	64.0-91.0	30 (75)	61.0-89.0	.5637
20. Help low milk supply	31 (78)	64.0-91.0	30 (75)	61.0-89.0	.7055
	***M* (*SD*)**	**95% CI for *M***	***M* (*SD*)**	**95% CI for *M***	
Total correct	12.9 (2.6)	12.0-13.7	16.7 (2.0)	16.1-17.3	<.0001

Abbreviation: NE, not estimable.
[a]For individual items, p value calculated with McNemar's chi-square test. For total correct, p value calculated with paired t test. Significant at $p < .05$.

Copyright © 2021 McLeod et al. This is an open-access publication distributed under the terms of the Creative Commons Attribution license.

Table 2. Differences Between Pre- and Post-BABA Scores for Students ($N = 40$)

BABA Item	Pre Median (IQR)	Pre 95% CI	Post Median (IQR)	Post 95% CI	p[a]
Confident addressing concerns	2 (2-3)		4 (3-4)		<.0001
Confidence in medication safety	3 (2-4)		4 (3-5)		<.0001
Confident in helping breastfeed	2 (1-3)		4 (3-4)		<.0001
Not embarrassed about breastfeeding in front of	5 (4-5)		5 (5-5)		.3594
Not embarrassed to help breastfeed	5 (4-5)		5 (4-5)		.0005
	***M* (*SD*)**		***M* (*SD*)**		
BABA total score	16.1 (3.6)	14.9-17.2	20.6 (2.8)	19.6-21.5	<.0001

Abbreviations: BABA, Breastfeeding Attitudes and Behavior Assessment; IQR, interquartile range.
[a]For individual items, p value calculated with Wilcoxon rank sum test. For total score, p value calculated with paired t test. Significant at $p < .05$.

Discussion

Learners can be uncomfortable working with newly breastfeeding mothers on their breastfeeding issues, which can negatively impact the mothers' breastfeeding experience and duration.[28] Although the efficacy of an SP encounter as an effective learning tool has been well established,[29] obtaining a number of breastfeeding dyads for these types of learning encounters is time and cost prohibitive.[23,30,31] Therefore, in an effort to provide a more accessible opportunity for breastfeeding education, we created three videos to train learners in two different settings. Students showed a statistically significant increase in breastfeeding knowledge and self-perceived comfort and confidence related to breastfeeding, and residents increased their knowledge and confidence.

As we reflect on this resource, there are a couple of changes we would recommend other facilitators to make as they implement this resource in their own context. For example, we suggest that if the videos are used during a formal teaching session, facilitators should consider adding an additional 15-30 minutes to allow for even more group discussion and a greater review of the learners' observations in the checklists. If there is enough time to review specific parts of the videos that may have been either subtle or missed, learners will be better primed to observe these moments when they interact with breastfeeding mothers.

We also suggest that immediately following the videos, facilitators make an effort to have a live breastfeeding dyad available for the group in order to evaluate to cement the knowledge gained

Table 3. Pre- and Postassessment Knowledge Test Scores for Residents ($N = 25$)

Questionnaire Item	Pre No. (%)	Pre 95% CI for %	Post No. (%)	Post 95% CI for %	p[a]
1. Enhance milk intake	8 (32)	12.3-51.7	24 (96)	87.7-100.0	.0002
2. Skin-to-skin benefits	20 (80)	63.1-96.9	24 (96)	87.7-100.0	.1025
3. Effective way break latch	9 (36)	15.8-56.2	24 (96)	87.7-100.0	.0001
4. Sore nipples indicate	14 (56)	35.1-76.9	23 (92)	80.6-100.0	.0067
5. Infant mouth assessment	7 (28)	9.1-46.9	21 (84)	68.6-99.4	.0002
6. Preterm at risk jaundice	19 (76)	58.0-94.0	23 (92)	80.6-100.0	.1573
7. Late hunger sign	2 (8)	0.0-19.4	20 (80)	63.1-96.9	<.0001
8. Milk supply promotion	22 (88)	74.3-100.0	23 (92)	80.6-100.0	.6547
9. Feed schedules	18 (72)	53.1-90.9	21 (84)	68.6-99.4	.2568
10. Adequate milk intake signs	10 (40)	19.4-60.6	10 (40)	19.4-60.6	1.0000
11. Recommended birth control	18 (72)	53.1-90.9	22 (88)	74.3-100.0	.1025
12. Confirm adequate milk	25 (100)	NE	24 (96)	87.7-100.0	NE
13. Good latch technique	21 (84)	68.6-99.4	20 (80)	63.1-96.9	.7055
14. Prevent hypoglycemia	19 (76)	58.0-94.0	20 (80)	63.1-96.9	.5637
15. Baby-friendly concepts	14 (56)	35.1-76.9	23 (92)	80.6-100.0	.0067
16. Sore nipple source	22 (88)	74.3-100.0	23 (92)	80.6-100.0	.6547
17. Hospital policy interfere breastfeeding	13 (52)	31.0-73.0	19 (76)	58.0-94.0	.0143
18. Delay breastfeeding initiation	13 (52)	31.0-73.0	14 (56)	35.1-76.9	.6547
19. When to supplement	18 (72)	53.1-90.9	17 (68)	48.3-87.7	.5637
20. Help low milk supply	18 (72)	53.1-90.9	18 (72)	53.1-90.9	1.0000
	***M* (*SD*)**	**95% CI for *M***	***M* (*SD*)**	**95% CI for *M***	
Total correct	12.4 (2.1)	11.5-13.3	17.2 (1.7)	16.5-17.9	<.0001

Abbreviation: NE, not estimable.
[a]For individual items, p value calculated with McNemar's chi-square test. For total correct, p value calculated with paired t test. Significant at $p < .05$.

Copyright © 2021 McLeod et al. This is an open-access publication distributed under the terms of the Creative Commons Attribution license.

Table 4. Differences Between Pre- and Post-BABA Test Scores for Residents ($N = 25$)

	Pre		Post		
**BABA Item**	**Median (IQR)**	**95% CI**	**Median (IQR)**	**95% CI**	p^a
Confident addressing concerns	3 (2-3)		4 (3-4)		.0004
Confidence in medication safety	3 (2-4)		4 (3-4)		.0649
Confident in helping breastfeed	2 (1-3)		4 (3-4)		<.0001
Not embarrassed about breastfeeding in front of	5 (5-5)		5 (5-5)		1.0000
Not embarrassed to help breastfeed	5 (4-5)		5 (4-5)		.5078
	***M* (SD)**		***M* (SD)**		
BABA total score	17.6 (3.5)	16.2-19.1	19.2 (3.8)	17.6-20.7	.0818

Abbreviation: BABA, Breastfeeding Attitudes and Behavior Assessment; IQR, interquartile range.
[a]For individual items, p value calculated with Wilcoxon rank sum test. For total score, p value calculated with paired t test.
Significant at $p < .05$.

in watching the videos. This would also allow learners to ask follow-up questions should they have any. Alternatively, if a breastfeeding mother is unavailable, the facilitator might want to have the group reflect on the video exercise to see if there are any remaining questions about proper breastfeeding.

There are several limitations that should be considered when using these videos to teach learners about breastfeeding. First, some of the concepts presented in the videos would have been better taught using a live breastfeeding mother. However, the videos may highlight some concepts better than others as a result of having a filmed, rather than live, mother. Second, because of time and schedule constraints, residents watched these videos in one afternoon, rather than over multiple sessions, which may have further internalized their knowledge and skills regarding breastfeeding. Students learners watched the videos on their own time and did not have the benefit of added learning by group discussion of key points. Finally, the BABA confidence and comfort scores were self-assessments. As a result, learners could incorrectly under- or overestimate their skill levels. Although the assessments were based on questionnaires with ample validity evidence, the final BABA in this curriculum was self-created and unpiloted prior to its use.

Future work in assisting learners with breastfeeding knowledge, comfort, and confidence may consider using other forms of data to assess comfort levels. For example, we recommend having an outside observer evaluate learners' comfort using observation of learners' body language as they help a breastfeeding mother. This suggestion is based on research that indicates physicians' reactions to breastfeeding impacts mothers' feelings about breastfeeding.[32] We also recommend supervising learners while they interact with breastfeeding mothers and interviewing breastfeeding mothers after learners have been exposed to this curriculum to reinforce their learning.

Appendices

A. Instructor Guide.docx
B. BABA Test.docx
C. Knowledge Test.docx
D. Sore Nipples Checklist.docx
E. Jaundice Checklist.docx
F. Perceived Low Milk Supply Checklist.docx
G. Sore Nipples.mp4
H. Jaundice.mp4
I. Perceived Low Milk Supply.mp4
J. Knowledge Test Answers.docx
K. Sore Nipples Checklist Answers.pdf
L. Jaundice Checklist Answers.pdf
M. Perceived Low Milk Supply Checklist Answers.pdf

All appendices are peer reviewed as integral parts of the Original Publication.

Kathryn McLeod, MD, IBCLC: Professor, Department of Pediatrics, Medical College of Georgia at Augusta University; ORCID: https://orcid.org/0000-0002-6870-4392

Jennifer Waller, PhD: Professor, Department of Population Health Sciences, Medical College of Georgia at Augusta University; ORCID: https://orcid.org/0000-0003-4066-4541

Tasha R. Wyatt, PhD: Associate Professor, Educational Innovation Institute, Medical College of Georgia at Augusta University; ORCID: https://orcid.org/0000-0002-0071-5298

Disclosures
None to report.

Funding/Support
None to report.

Copyright © 2021 McLeod et al. This is an open-access publication distributed under the terms of the Creative Commons Attribution license.

Informed Consent
All identifiable persons in this resource have granted their permission.

Ethical Approval
Reported as not applicable.

References

1. Chowdhury R, Sinha B, Sankar MJ, et al. Breastfeeding and maternal health outcomes: a systematic review and meta-analysis. *Acta Paediatr.* 2015;104(S467):96-113. https://doi.org/10.1111/apa.13102
2. Sankar MJ, Sinha B, Chowdhury R, et al. Optimal breastfeeding practices and infant and child mortality: a systematic review and meta-analysis. *Acta Paediatr.* 2015;104(S467):3-13. https://doi.org/10.1111/apa.13147
3. Chantry CJ, Howard CR, Auinger P. Full breastfeeding duration and associated decrease in respiratory tract infection in US children. *Pediatrics.* 2006;117(2):425-432. https://doi.org/10.1542/peds.2004-2283
4. Feldman-Winter L, Barone L, Milcarek B, et al. Residency curriculum improves breastfeeding care. *Pediatrics.* 2010;126(2):289-297. https://doi.org/10.1542/peds.2009-3250
5. Anchondo I, Berkeley L, Mulla ZD, et al. Pediatricians', obstetricians', gynecologists', and family medicine physicians' experiences with and attitudes about breast-feeding. *South Med J.* 2012;105(5):243-248. https://doi.org/10.1097/SMJ.0b013e3182522927
6. Breastfeeding Report Card. Centers for Disease Control and Prevention. Updated September 17, 2020. Accessed October 19, 2020. https://www.cdc.gov/breastfeeding/data/reportcard.htm
7. Lowe NK. The Surgeon General's call to action to support breastfeeding. *J Obstet Gynecol Neonatal Nurs.* 2011;40(4): 387-389. https://doi.org/10.1111/j.1552-6909.2011.01266.x
8. Ogbuanu CA, Probst J, Laditka SB, Liu J, Baek J, Glover S. Reasons why women do not initiate breastfeeding: a southeastern state study. *Womens Health Issues.* 2009;19(4):268-278. https://doi.org/10.1016/j.whi.2009.03.005
9. Renfrew MJ, McFadden A, Dykes F, et al. Addressing the learning deficit in breastfeeding: strategies for change. *Matern Child Nutr.* 2006;2(4):239-244. https://doi.org/10.1111/j.1740-8709.2006.00068.x
10. Taveras EM, Li R, Grummer-Strawn L, et al. Opinions and practices of clinicians associated with continuation of exclusive breastfeeding. *Pediatrics.* 2004;113(4):e283-e290. https://doi.org/10.1542/peds.113.4.e283
11. Frank JR, Snell LS, Ten Cate O, et al. Competency-based medical education: theory to practice. *Med Teach.* 2010;32(8):638-645. https://doi.org/10.3109/0142159X.2010.501190
12. Haughwout JC, Eglash AR, Plane MB, Mundt MP, Fleming MF. Improving residents' breastfeeding assessment skills: a problem-based workshop. *Fam Pract.* 2000;17(6):541-546. https://doi.org/10.1093/fampra/17.6.541
13. Buchwald J. The first pelvic examination: helping students cope with their emotional reactions. *J Med Educ.* 1979;54(9):725-728. https://doi.org/10.1097/00001888-197909000-00005
14. Vontver L, Irby D, Rakestraw P, Haddock M, Prince E, Stenchever M. The effects of two methods of pelvic examination instruction on student performance and anxiety. *J Med Educ.* 1980;55(9): 778-785. https://doi.org/10.1097/00001888-198009000-00007
15. Breastfeeding Residency Curriculum: faculty implementation guide. American Academy of Pediatrics. Accessed January 4, 2020. https://www.aap.org/en-us/advocacy-and-policy/aap-health-initiatives/Breastfeeding/Pages/Evaluation-Tools.aspx
16. Getting started with breastfeeding. Stanford Medicine. Accessed January 1, 2018. https://med.stanford.edu/newborns/professional-education/breastfeeding.html
17. Aby J, Schiller J. Newborn nursery teaching attending guide: Module 5. Newborn feeding. *MedEdPORTAL*. 2012;8:9157. https://doi.org/10.15766/mep_2374-8265.9157
18. Kelly S, Vince R. Residents as teachers: a unique approach to teaching infant feeding and normal growth to pediatric residents in the continuity clinic setting. *MedEdPORTAL*. 2015;11:10118. https://doi.org/10.15766/mep_2374-8265.10118
19. McLeod K, Whitaker R, Thomas A, et al. A qualitative comparison of feedback from a breastfeeding OSCE exercise for pediatric second year residents. *Poster presented at: Academy of Breastfeeding Medicine Annual International Meeting*; November 5, 2011; Miami, FL.
20. McLeod K, McCullen T, Wilson D, Wood S, Richardson D. Interdisciplinary development of a breastfeeding curriculum for first and second year pediatric residents. *Poster presented at: Academy of Breastfeeding Medicine Annual International Meeting*; 2010; San Francisco, CA.
21. McLeod K, Whitaker R, Palladino C, Thomas A. The effect of a new breastfeeding curriculum on resident confidence, comfort and skills. Oral presentation at: American Academy of Pediatrics National Conference and Exhibition, Section on Breastfeeding; 2011; Boston, MA.
22. Jha V, Setna Z, Al-Hity A, Quinton ND, Roberts TE. Patient involvement in teaching and assessing intimate examination skills: a systematic review. *Med Educ.* 2010;44(4):347-357. https://doi.org/10.1111/j.1365-2923.2009.03608.x
23. Adamo G. Simulated and standardized patients in OSCEs: achievements and challenges 1992-2003. *Med Teach.* 2003;25(3):262-270, https://doi.org/10.1080/0142159031000100300
24. Gary AJ, Birmingham EE, Jones LB. Improving breastfeeding medicine in undergraduate medical education: a student survey and extensive curriculum review with suggestions for improvement. *Educ Health (Abingdon).* 2017;30(2):163-168. https://doi.org/10.4103/efh.EfH_180_15

Copyright © 2021 McLeod et al. This is an open-access publication distributed under the terms of the Creative Commons Attribution license.

25. Hillenbrand KM, Larsen PG. Effect of an educational intervention about breastfeeding on the knowledge, confidence, and behaviors of pediatric resident physicians. *Pediatrics.* 2002;110(5):e59. https://doi.org/10.1542/peds.110.5.e59

26. McLeod K, Elam R, Palladino C, Thomas A. The effect of a breastfeeding OSCE curriculum on resident confidence and comfort. Presented at: American Academy of Pediatrics National Conference and Exhibition, Section on Breastfeeding; 2012; New Orleans, LA.

27. McLeod K, Thomas A, Whitaker R, Richardson D. Assessment of a breastfeeding curriculum intervention for first and second year pediatric residents. Presented at: Southern Group on Educational Affairs (SGEA) Regional Meeting; 2011; Houston, TX.

28. Radzyminski S, Callister LC. Health professionals' attitudes and beliefs about breastfeeding. *J Perinat Educ.* 2015;24(2):102-109. https://doi.org/10.1891/1058-1243.24.2.102

29. Cleland JA, Abe K, Rethans JJ. The use of simulated patients in medical education: AMEE Guide no 42. *Med Teach.* 2009;31(6): 477-486. https://doi.org/10.1080/01421590903002821

30. Calvert MJ, Freemantle N. Cost-effective undergraduate medical education? *J R Soc Med.* 2009;102(2):46-48. https://doi.org/10.1258/jrsm.2008.080353

31. Bosse HM, Nickel M, Huwendiek S, Schultz JH, Nikendei C. Cost-effectiveness of peer role play and standardized patients in undergraduate communication training. *BMC Med Educ.* 2015;15:183. https://doi.org/10.1186/s12909-015-0468-1

32. DiGirolamo AM, Grummer-Strawn LM, Fein SB. Do perceived attitudes of physicians and hospital staff affect breastfeeding decisions? *Birth.* 2003;30(2):94-100. https://doi.org/10.1046/j.1523-536X.2003.00227.x

Received: June 24, 2020
Accepted: February 1, 2021
Published: April 1, 2021

Copyright © 2021 McLeod et al. This is an open-access publication distributed under the terms of the Creative Commons Attribution license.
